# The association of partial pressures of oxygen and carbon dioxide with neurological outcome after out-of-hospital cardiac arrest: an explorative International Cardiac Arrest Registry 2.0 study

**DOI:** 10.1186/s13049-020-00760-7

**Published:** 2020-07-14

**Authors:** Florian Ebner, Richard R. Riker, Zana Haxhija, David B. Seder, Teresa L. May, Susann Ullén, Pascal Stammet, Karen Hirsch, Sune Forsberg, Allison Dupont, Hans Friberg, John A. McPherson, Eldar Søreide, Josef Dankiewicz, Tobias Cronberg, Niklas Nielsen

**Affiliations:** 1 Lund University, Helsingborg Hospital, Department of Clinical Sciences Lund, Anesthesia and Intensive Care, Charlotte Yhlens Gata 10, S-251 87 Helsingborg, Sweden; 2 Department of Critical Care Services, Maine Medical Center, Portland, ME USA; 3 Clinical Studies Sweden, Skane University Hospital, Lund, Sweden; 4 Medical and Health Directorate, National Fire and Rescue Corps, Luxembourg City, Luxembourg; 5 Stanford Neurocritical Care Program, Department of Neurology and Neurological Sciences, Stanford University School of Medicine, Stanford, California USA; 6 Department of Intensive Care, Norrtälje Hospital, Center for Resuscitation,Karolinska Institute, Solna, Sweden; 7 Department of Cardiology, Northeast Georgia Medical Center, Gainesville, GA USA; 8 Department of Clinical Sciences, Anesthesiology and Intensive Care, Lund University, Skane University Hospital, Malmö, Sweden; 9 Vanderbilt University Medical Center, Nashville, TN USA; 10 Critical Care and Anaesthesiology Research Group, Stavanger University Hospital, Stavanger, Norway; 11 Department of Clinical Medicine, University of Bergen, Bergen, Norway; 12 Department of Clinical Sciences Lund, Cardiology, Lund University, Skane University Hospital, Lund, Sweden; 13 Department of Clinical Sciences Lund, Neurology, Lund University, Skane University Hospital, Lund, Sweden

**Keywords:** Oxygen, Carbon dioxide, Out-of-hospital cardiac arrest, Brain anoxia-ischemia, Cardio-pulmonary resuscitation, Critical care outcomes

## Abstract

**Background:**

Exposure to extreme arterial partial pressures of oxygen (PaO_2_) and carbon dioxide (PaCO_2_) following the return of spontaneous circulation (ROSC) after out-of-hospital cardiac arrest (OHCA) is common and may affect neurological outcome but results of previous studies are conflicting.

**Methods:**

Exploratory study of the International Cardiac Arrest Registry (INTCAR) 2.0 database, including 2162 OHCA patients with ROSC in 22 intensive care units in North America and Europe. We tested the hypothesis that exposure to extreme PaO_2_ or PaCO_2_ values within 24 h after OHCA is associated with poor neurological outcome at discharge. Our primary analyses investigated the association between extreme PaO_2_ and PaCO_2_ values, defined as hyperoxemia (PaO_2_ > 40 kPa), hypoxemia (PaO_2_ < 8.0 kPa), hypercapnemia (PaCO_2_ > 6.7 kPa) and hypocapnemia (PaCO_2_ < 4.0 kPa) and neurological outcome. The secondary analyses tested the association between the exposure combinations of PaO_2_ > 40 kPa with PaCO_2_ < 4.0 kPa and PaO_2_ 8.0–40 kPa with PaCO_2_ > 6.7 kPa and neurological outcome. To define a cut point for the onset of poor neurological outcome, we tested a model with increasing and decreasing PaO_2_ levels and decreasing PaCO_2_ levels. Cerebral Performance Category (CPC), dichotomized to good (CPC 1–2) and poor (CPC 3–5) was used as outcome measure.

**Results:**

Of 2135 patients eligible for analysis, 700 were exposed to hyperoxemia or hypoxemia and 1128 to hypercapnemia or hypocapnemia. Our primary analyses did not reveal significant associations between exposure to extreme PaO_2_ or PaCO_2_ values and neurological outcome (*P* = 0.13–0.49). Our secondary analyses showed no significant associations between combinations of PaO_2_ and PaCO_2_ and neurological outcome (*P* = 0.11–0.86). There was no PaO_2_ or PaCO_2_ level significantly associated with poor neurological outcome. All analyses were adjusted for relevant co-variates.

**Conclusions:**

Exposure to extreme PaO_2_ or PaCO_2_ values in the first 24 h after OHCA was common, but not independently associated with neurological outcome at discharge.

## Introduction

Admission to hospital as well as 30-day survival after out of hospital cardiac arrest (OHCA) has increased in recent years and most 30-day survivors after OHCA are discharged with good neurological function [1]. Despite these advances, the proportion of patients dying after hospital admission is more than 50 % and the major causes are the primary ischemic cerebral injury sustained during the no-flow time of the OHCA and the additional secondary cerebral reperfusion injury that commences at return of spontaneous circulation (ROSC) [2, 3]. Reperfusion entails increased reactive oxygen species (ROS) production, mitochondrial dysfunction and apoptosis, and thus, exacerbates the detrimental consequences of the OHCA [3]. Targeted temperature management (TTM) has been suggested as an intervention to attenuate these effects but studies are inconclusive and current studies indicate varying use internationally [4–8]. Recent data suggest that elevated arterial partial pressure of carbon dioxide (PaCO_2_), hypercapnemia, might improve neurological outcome after OHCA. Possible underlying mechanisms include decreased cerebral vascular resistance (CVR), increased cerebral blood flow (CBF), modulation of inflammatory processes and anti-convulsive properties [9–16]. In contrast to hypercapnemia, low PaCO_2_, hypocapnemia, increases CVR, decreases CBF, reduces oxygen delivery (CDO_2_) and is associated with poor outcome [10, 16–19]. Low arterial partial pressure of oxygen (PaO_2_), hypoxemia, is the primary source of neuronal injury occurring during the OHCA and a determinant of neurological outcome [3, 20]. Elevated PaO_2_, hyperoxemia, has also been associated with poor neurological outcome, possibly due to increased lipid oxidation, production of ROS, mitochondrial damage and reduced CBF [3, 21–23]. The association of combinations of extreme PaO_2_ and PaCO_2_ values after OHCA with outcome have less frequently been studied, but the combination of moderate hypercapnemia and mild hyperoxemia was association with improved neurological outcome in one study [24]. Overall study results are inconsistent and other investigations trying to confirm the protective or harmful associations of exposure to extreme PaO_2_ and PaCO_2_ values with neurological outcome were unable to do so [25–27]. Moreover, the available studies differ in methodology, inclusion criteria and may lack sufficient power. Therefore, we conducted this study of the International Cardiac Arrest Registry (INTCAR) 2.0 database to investigate the association between exposure to extreme PaCO_2_ and PaO_2_ values and neurological outcome at hospital discharge in a large cohort of adult, unconscious patients with sustained ROSC after OHCA.

## Methods

INTCAR 2.0 is an international multicenter database including cardiac arrest patients admitted to intensive care units (ICU) at 22 medical centers in the United States and Europe. The present investigation of the INTCAR 2.0 database included prospectively collected cardiac arrest and treatment data from adult (≥18 years of age), unconscious (GCS < 8), OHCA patients with sustained ROSC. All patients in this study received TTM treatment and were admitted between 2008 and 2018. Patient data collected in the database was anonymized and OHCA data was reported according to the Utstein-style protocol [28]. Ethical committees in each participating country approved the data collection and analysis. Informed consent was either waived or obtained from all participants or relatives according to national and local standards, in line with the Helsinki declaration. Reporting of our analyses was guided by the STROBE recommendations [29].

### Definition of PaO_2_ and PaCO_2_ groups and data registration

In the INTCAR 2.0 protocol, extreme PaO_2_ or PaCO_2_ exposure thresholds were defined as PaO_2_ > 40 kPa, PaO_2_ < 8.0 kPa, PaCO_2_ > 6.7 kPa and PaCO_2_ < 4.0 kPa. Exposure to one or more extreme values during the first 24 h after ROSC was registered in a dichotomous manner (yes/no). The PaO_2_ and PaCO_2_ thresholds were aligned with previous studies [17, 21, 22]. Additionally, the single highest and lowest PaO_2_ values and the lowest PaCO_2_ value during the first 24 h after ROSC were documented, regardless of exposure level. In total 7 data-points (4 PaO_2_ and 3 PaCO_2_ data-points) were collected per patient. For the purpose of this study we divided patients according to their extreme PaO_2_ or PaCO_2_ value exposure into four groups defined by the extreme values in the INTCAR 2.0 protocol; hyperoxemia (PaO_2_ > 40 kPa), hypoxemia (PaO_2_ < 8.0 kPa), hypercapnemia (PaCO_2_ > 6.7 kPa) and hypocapnemia (PaCO_2_ < 4.0 kPa). Patients not exposed to extreme values were classified as PaO_2_ and PaCO_2_ no-exposure (PaO_2_ 8.0–40 kPa and PaCO_2_ 4.0–6.7 kPa). Patients exposed to more than one extreme value were included in all exposure groups.

### Outcome

To better compare with previous analyses [21, 22, 27, 30], cerebral performance category (CPC) at discharge from hospital was chosen as primary outcome endpoint. After neurological assessment at hospital discharge by a trained health care professional OHCA patients were allocated to one of the five CPC categories, ranging from CPC1 (good cerebral performance/mild disability), CPC2 (moderate disability), CPC3 (severe disability), CPC4 (coma state) and CPC5 (brain death) [31, 32]. For this study we dichotomized outcome into good (CPC1 and 2) and poor (CPC3–5). Delayed outcomes, typically around 6 months after presentation, were also collected, by telephone interview or medical records.

In our primary analysis, we tested the association of exposure to extreme PaO_2_ or PaCO_2_ values with outcome. We conducted 8 analyses: 1.the hyperoxemia group was compared to the PaO_2_ no-exposure group and 2.to patients without hyperoxemia (no-hyperoxemia). The hypoxemia group was compared 3.to the PaO_2_ no-exposure group and 4.to patients not exposed to hypoxemia (no-hypoxemia). Patients in the hypercapnemia group were compared 5.to patients in the PaCO_2_ no-exposure group and 6.to patients without hypercapnemia exposure (no-hypercapnemia), while patients with hypocapnemia exposure were compared 7.to the PaCO_2_ no-exposure group and 8.to patients not exposed to hypocapnemia (no-hypocapnemia).

In previous studies, exposure to hyperoxemia, hypoxemia and hypocapnemia were associated with poor outcome while hypercapnemia was associated with good outcome [13, 18, 21, 22]. In our secondary analyses we therefore, a priori, defined exposure groups to investigate these findings and compared patients exposed to the combination of hyperoxemia with hypocapnemia to a PaO_2_ and PaCO_2_ no-exposure group, followed by a PaO_2_ no-exposure group with hypercapnemia compared to the PaO_2_ and PaCO_2_ no-exposure group. Subsequently, we designed regression models with ascending and descending PaO_2_ values from < 20 kPa to > 60 kPa and > 8.0 kPa to < 5.0 kPa to define a possible threshold for the onset of the association of hyperoxemia or hypoxemia and poor outcome. We also designed a regression model for the onset of the association of hypocapnemia and poor outcome with descending PaCO_2_ values from > 4.0 kPa to < 3.5 kPa.

### Sensitivity analyses

Sensitivity analyses were performed for our primary analyses with all double exposed patients (hyperoxemia and hypoxemia or hypercapnemia and hypocapnemia) removed. Furthermore, we performed sensitivity analyses of our primary analyses, replacing outcome at discharge with long-term outcome at 6-month follow-up.

### Statistical analysis

Proportions are presented as numbers and percentages and continuous variables as means with standard deviations (SD) or medians with interquartile ranges (IQR). Logistic regression analysis was used to assess the association between PaO_2_ and PaCO_2_ and neurological outcome at discharge. For the ascending analysis the odds ratio (OR) above the threshold was compared to the OR under the threshold, while for the descending analyses the OR under the threshold was compared to the OR above the threshold. All analyses were adjusted for pre-specified, and OHCA relevant co-variates: age (years), sex (male/female), previous chronic heart failure (yes/no), previous chronic obstructive pulmonary disease (COPD) (yes/no), cardiac arrest witnessed (yes/no), bystander cardiopulmonary resuscitation (yes/no), initial rhythm shockable (yes/no), time to ROSC, admission GCS-M 1 vs 2–5, circulatory shock, TTM-treatment (low, 32–34 degrees Celsius (°C) versus high, 35–37 °C) and pH on admission as fixed effects and treatment site as a random effect. None of the independent variables included in our models were highly correlated. We conducted two-sided tests and considered a *P*-value < 0.05 as significant. We included patients with complete data (PaO_2_ and/or PaCO_2_ and CPC at discharge registered) in the primary and secondary analyses. However, for our long term outcome sensitivity analysis, we imputed missing outcome (last observation (CPC at discharge) carried forward). Analyses were conducted using R: A language and environment for statistical Computing (version 3.3.3 R Foundation for Statistical Computing, Vienna, Austria) [33].

## Results

**Fig. 1:**
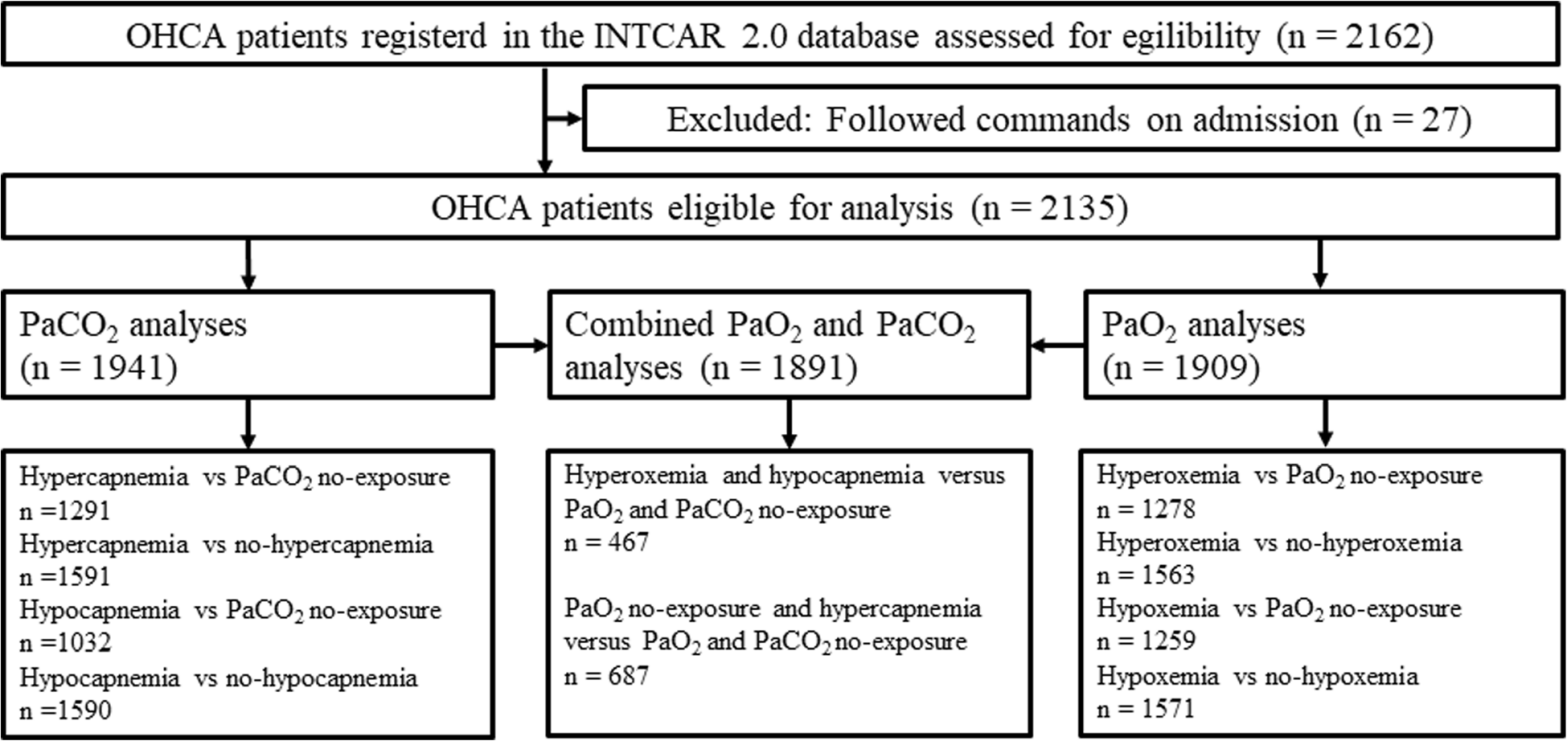
Patient selection pathway. OHCA = out-of-hospital cardiac arrest, *n* = number, PaO_2_ = arterial partial pressure of oxygen, PaCO_2_ arterial partial pressure of carbon dioxide, vs = versus

The INTCAR 2.0 database included 2162 OHCA patients who were assessed for eligibility. Of this cohort, we excluded 27 patients who experienced OHCA but were not unconscious on admission. The remaining 2135 patients were included in our final analysis (Fig. 1). Baseline data for this group is displayed in Table 1. Baseline data for the different PaO_2_ and PaCO_2_ exposure groups are displayed in the Additional File, Tables 1 and 2. Six hundred eighteen (28.9%) patients experienced a good outcome and 1517 (71.1%) a poor outcome. Eight hundred twenty-eight (38.8%) patients were alive at discharge, while 1307 (61.2%) were dead. At 6-month follow-up the outcome of 634 (29.7%) patients was good, whereas 1501 (70.3%) patients had a poor outcome in the cohort with imputed data. The cohort without imputation showed a good outcome in 450 (24.3%) and a poor outcome in 1400 (75.7%) patients. All patients received TTM treatment during the first 24 h after ROSC, 1673 (78.4%) to target temperature 32–34 °C and 462 (21.6%) to 35–37 °C. Three hundred and fifty-seven (18.7%) patients were exposed to hyperoxemia, 343 (17.9%) patients to hypoxemia and 76 (3.9%) to both, while 670 (34.5%) patients experienced hypercapnemia, 458 (23.6%) hypocapnemia and 222 (11.4%) both. During the first 24 h after OHCA, median highest PaO_2_ was 25.7 (IQR 18.5–38.1) kPa, median lowest PaO_2_ was 10.0 (IQR 8.1–12.7) kPa and median lowest PaCO_2_ was 4.3 (IQR 3.7–4.9) kPa.

**Table 1:** Baseline characteristics of patients included in the PaO_2_ and PaCO_2_ analyses, *n* = 2135

Demographic characteristic	Value
Age in years, mean (SD)	61.09 (15.9)
Male sex, n (%)	1432 (67.1)
**Medical history**	
Previous myocardial infarction n (%)	370 (17.3)
Chronic heart failure n (%)	367 (17.2)
COPD n (%)	344 (16.1)
Cerebro vascular disease n (%)	196 (9.2)
Diabetes mellitus n (%)	521 (24.4)
Obesity n (%)	268 (15.3)
**Cardiac arrest characteristic**	
Witnessed cardiac arrest n (%)	1591 (75.6)
Bystander CPR n (%)	1385 (65.5)
Bystander defibrillation n (%)	123 (5.8)
Initial rhythm shockable n (%)	1022 (50.0)
Time to ROSC (min), median (IQR)	29 (21–48)
**Characteristic on arrival**	
Sedated on arrival n (%)	437 (21.7)
GCS Motor 1 n (%)	1544 (79.4)
Circulatory shock on admission n (%)	902 (44.2)
Admission pH, median (IQR)	7.2 (7.1–7.3)
Admission lactate, mmol/l, median (IQR)	6.4 (3.2–10.2)
Bicarbonate on admission, mmol/l, median (IQR)	18.0 (14.5–21.0)

*n* number, *SD* standard deviation, *IQR* interquartile range, *%* percent, *mmol/l* millimole per liter, *CPR* cardio pulmonary resuscitation, *ROSC* return of spontaneous circulation, *COPD* chronic obstructive pulmonary disease, *GCS* Glasgow coma scale, *PaO*_*2*_ arterial partial pressure of oxygen, *PaCO*_*2*_ arterial partial pressure of carbon dioxide, all % are presented as valid percent

In our primary analyses we found, after adjustment, neither hyperoxemia nor hypoxemia exposure in the first 24 h after ROSC to be associated with poor neurological outcome (all analyses, *P* = 0.13–0.44) (Table 2). Exposure to hyper- or hypocapnemia during the first 24 h after ROSC was also not associated with poor outcome (all analyses, *P* = 0.18–0.49) (Table 2).

**Table 2:** Association of exposure to extreme PaO_2_ and PaCO_2_ values with poor neurological outcome

Analysis	OR	95% CI	***P***-Value
Hyperoxemia versus PaO_2_ no-exposure	1.33	0.92–1.92	0.13
Hyperoxemia versus no-hyperoxemia	1.25	0.88–1.77	0.22
Hypoxemia versus PaO_2_ no-exposure	1.26	0.87–1.82	0.22
Hypoxemia versus no-hypoxemia	1.15	0.81–1.64	0.44
Hypercapnemia versus PaCO_2_ no-exposure	0.89	0.64–1.24	0.49
Hypercapnemia versus no-hypercapnemia	0.86	0.64–1.15	0.31
Hypocapnemia versus PaCO_2_ no-exposure	1.28	0.90–1.83	0.18
Hypocapnemia versus no-hypocapnemia	1.23	0.91–1.66	0.18

*OR* odds ratio, *95% CI* 95% confidence interval, *PaO*_*2*_ arterial partial pressure of oxygen, *PaCO*_*2*_ arterial partial pressure of carbon dioxide. Hyperoxemia = PaO_2_ > 40 kPa, Hypoxemia = PaO_2_ < 8.0 kPa, Hypercapnemia = PaCO_2_ > 6.7 kPa, Hypocapnemia = PaCO_2_ < 4.0 kPa. PaO_2_ no-exposure = 8.0–40 kPa PaCO_2_ no-exposure = 4.0–6.7 kPa

**Fig. 2:**
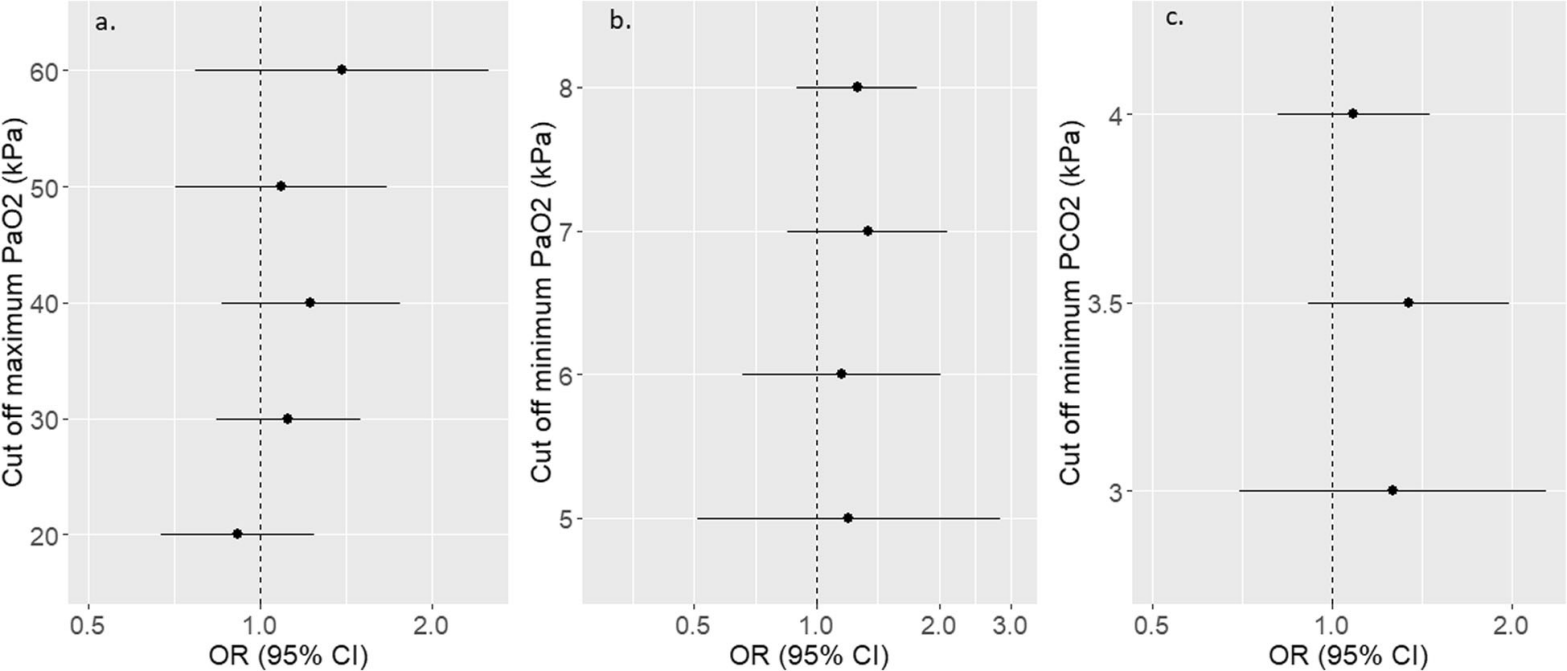
**a**-**c**. Forest plot showing the adjusted ORs (bullet points) with 95% CI (horizontal lines) for poor neurological outcome (CPC 3–5) across ascending PaO_2_ cut-off points (**a**), descending PaO_2_ cut-off points (**b**) and descending PaCO_2_ cut-off points (**c**). ORs and CIs are presented on a logarithmic scale. For (**a**), OR above 1.0 indicates worse outcome above the PaO_2_ threshold. For (**b**) and (**c**), OR above 1.0 indicates worse outcome under the PaO_2_ or PaCO_2_ threshold. OR = Odds ratio, 95% CI = 95% confidence interval, CPC = cerebral performance category, PaO_2_ = arterial partial pressure of oxygen, PaCO_2_ = arterial partial pressure of carbon dioxide. All analyses were adjusted for co-variates

In our secondary analysis the outcomes for patients exposed to the combination of hyperoxemia with hypocapnemia showed no association with poor neurological outcome (*P* = 0.11, Table 3). The exposure combination of hypercapnemia with PaO_2_ no-exposure was also not associated with poor outcome (*P* = 0.86, Table 3). Figure 2a and b depict the adjusted OR with 95% CIs for poor neurological outcome across ascending and descending PaO_2_ cut off values. Figure 2c shows the adjusted OR with 95% CIs for poor neurological outcome across descending PaCO_2_ cut off values. We did not detect a significant threshold value for the onset of an association with poor outcome in any of these three analyses.

**Table 3:** Association of PaO_2_ and PaCO_2_ combinations with poor neurological outcome

Analysis	OR	95% CI	***P***-Value
Hyperoxemia and hypocapnemia versus PaO_2_ and PaCO_2_ no-exposure	1.67	0.89–3.14	0.11
PaO_2_ no-exposure and hypercapnemia versus PaO_2_ and PaCO_2_ no-exposure	0.96	0.63–1.48	0.86

*OR* odds ratio, *95% CI* 95% confidence interval, *PaO*_*2*_ arterial partial pressure of oxygen, *PaCO*_*2*_ arterial partial pressure of carbon dioxide. Hyperoxemia = PaO_2_ > 40 kPa, Hypoxemia = PaO_2_ < 8.0 kPa, Hypercapnemia = PaCO_2_ > 6.7 kPa, Hypocapnemia = PaCO_2_ < 4.0 kPa. PaO_2_ no-exposure = 8.0-40 kPa, PaCO_2_ no-exposure = 4.0–6.7 kPa

### Sensitivity analyses

The results of the sensitivity analysis with all double exposed patients (hyperoxemia and hypoxemia or hypercapnemia and hypocapnemia) removed were similar to the results of our primary analyses (*P* = 0.07–0.29) (Additional File, Table 3). Replacing outcome at discharge with long term outcome in our primary analyses did not change our results significantly, neither in the dataset without imputed outcome measures (*P* = 0.14–0.89) nor in the dataset with missing outcome measures imputed (*P* = 0.13–0.59) (Additional File, Table 4 and 5).

### Missing data

244 patients had one or more PaO_2_ or PaCO_2_ data points missing. Comparing this group with the group of patients with complete PaO_2_ and PaCO_2_ data (*n* = 1891) showed similar values at baseline (Additional File, Table 6).

## Discussion

In this exploratory study testing the associations between exposure to extreme PaCO_2_ and PaO_2_ values and neurological outcomes at discharge of 2135 patients with OHCA, we found that exposure to extreme PaO_2_ and PaCO_2_ values was common, but not significantly associated with neurological outcome after adjusting for in the context of OHCA-relevant covariates. In our subsequent analyses, we did not show any significant associations of combinations of PaO_2_ and PaCO_2_ and poor neurological outcomes. Despite investigating PaO_2_ values to > 60 kPa and < 5.0 kPa and PaCO_2_ values to < 3.5 kPa in our ascending and descending cut-off point analyses, we did not identify a numerical threshold for the onset of the association of each variable with poor neurological outcome. These findings suggest that PaO_2_ and PaCO_2_ may not be directly associated with outcome after resuscitation from OHCA. Animal studies have shown worse neurological outcomes and increased neurological injury after exposure to hyperoxemia following resuscitation from cardiac arrest and indicate that hyperoxemia in the post cardiac arrest phase might be harmful [34]. These findings have been corroborated by retrospective observational human studies [21, 35, 36]. Moreover, a threshold for the onset of poor outcome has been proposed at 40 kPa [21]. Elmer et al. confirmed the previously suggested hyperoxemia threshold of 40 kPa for the onset of poor outcome but also showed that moderate hyperoxemia (PaO_2_ 13.5–39.9 kPa) was associated with lower SOFA scores at 24 h, indicating a possibly beneficial effect at these levels [37]. This finding was supported by a study of Helmerhorst et al. investigating 5258 cardiac arrest patients, displaying a U-shaped relationship between PaO_2_ and outcome and, although not significant, the lowest probability of in-hospital death between 13.6–40 kPa [38]. However, not all investigations support these results [27, 39], and studies are frequently of retrospective design, correct for different confounders and investigate mixed IHCA and OHCA cohorts.

A recent multi-center study of 280 patients across 6 hospitals in the United States by Roberts et al., sampled blood gases at 1 and 6 h after ROSC and found that early hyperoxemia was associated with poor outcome at discharge. The investigators also substantiated the suggested threshold for the onset of poor outcome at 40 kPa. We investigated comparable PaO_2_ levels in our study and although our results were not significant, the point estimates of our primary analyses indicate higher probabilities for poor outcome in the hyperoxemia group but also in the hypoxemia group. We did not identify a significant threshold for the onset of poor neurological outcome in our cut-off analysis but the lowest probability of poor outcome was in the group exposed to a PaO_2_ of up to 20 kPa which was similar to the risk ratio analysis by Roberts et al. Nevertheless, there are noteworthy differences between the investigations; our cohort was significantly larger than Roberts et al. and we included exclusively OHCA patients in order to increase homogeneity regarding cardiac arrest etiology. Furthermore, and most importantly, Roberts et al. sampled blood gases according to a prospective protocol over the first 6 h whereas our study evaluated the most extreme blood gas values during the first 24 h after ROSC.

Exposure to hypercapnemia or hypocapnemia in the post cardiac arrest phase is common [17, 18, 25, 40] and hypocapnemia has frequently been associated with poor outcome [17, 18, 41] while hypercapnemia exposure has been associated with poor outcome [17, 30, 38, 42], good outcome [12, 13, 18, 24] or no difference in outcome [25, 41]. In an analysis of 9176 adult OHCA patients in the ROC-network, Wang et al. showed that hypercapnemia at any time-point within the first 24 h after OHCA and hypocapnemia towards the end of the first 24 h was associated with increased in-hospital mortality. Our study employed the same cut-off levels for hypercapnemia and hypocapnemia as Wang et al., but the prevalence of hypercapnemia and hypocapnemia were lower in our analysis (34.5% versus 51.0 and 23.6% versus 30.6%, respectively). The overall in-hospital mortality of Wang et al. was similar to our proportion of patients with CPC5 at discharge (67.3% versus 61.2%), but we did not achieve significant results in our analyses, and somewhat contrary to the ROC-network analysis our point estimates indicate a lower probability for poor outcome in the group exposed to hypercapnemia. However, the studies are not entirely comparable; Wang et al. included significantly more patients and analyzed the first, last or any arterial blood gas measurement during the first 24 h of hospitalization, while our study analyzed the most extreme values within 24 h of ICU admission. Moreover, Wang et al. did not correct for in-hospital care such as induced hypothermia or physiological parameters as pH.

Considering the results of the studies investigating PaO_2_ or PaCO_2_, the exposure to combinations of extreme PaO_2_ and PaCO_2_ values might also be associated with neurological outcome. Vahersaalo et al. found in a cohort of 409 OHCA patients the combination of moderate hypercapnemia and mild hyperoxemia to be associated with improved neurological outcome. We investigated hypercapnemia in combination with PaO_2_ 8.0–40 kPa, but were not able to show an association with an improved outcome in this group. Treatment with induced hypothermia to 32–34 °C might influence CO_2_ solubility and represent a potential bias between analyses, but the 32–34 °C groups were of similar size in both studies (71% versus 78.4%). Nevertheless, there were significant differences, most notably, Vahersaalo et al. measured mean PaO_2_ and PaCO_2_ values in different ranges whereas we analyzed exposure to the most extreme values.

As shown above, studies investigating extreme PaO_2_ and PaCO_2_ value exposure after cardiac arrest differ in inclusion criteria and the time frame after ROSC, objectives and results. Moreover, short term variability in vascular tone and acid-balance due changes in the fraction of inspired oxygen (FiO_2_) or respiratory rate is commonly not accounted for [23, 43]. It seems also important to point out that the possible protective and harmful properties associated with exposure to extreme PaO_2_ and PaCO_2_ values are still of a largely hypothetical nature. Hypercapnemia increases CBF and might improve outcome by optimizing CBF and CDO_2_ after OHCA as suggested by Eastwood et al. [13, 15]. Hypercapnemia is also an effective anticonvulsant, suppressing neuronal activity in the central nervous system and potentially reducing neuronal metabolic demands following ROSC, but so far, hypercapnemia has failed to show an association with favorable EEG patterns after OHCA [9, 44–46]. The optimal dose of hypercapnemia in the post OHCA phase, if favorable, is not known. Two randomized controlled pilot-studies investigating high normal PaCO_2_ (5.8–6.0 kPa) and mild hypercapnemia (6.7–7.3 kPa) have used neuron specific enolase (NSE) as a surrogate marker of neuronal injury [13, 44]. NSE was significantly reduced in patients exposed to mild hypercapnemia, while high normal PaCO_2_ exposure was not associated with NSE levels after OHCA. Although, consistent high quality evidence is lacking, there are no indicators of harmful effects of controlled hypercapnemia exposure after OHCA [13, 25, 44]. However, the results of the present study conflict with results from previous investigations and support the need for further randomized trials [47, 48].

Neuronal metabolic failure due to hypoxemia during the no-flow period of the OHCA is the principal cause of cerebral damage, but also hyperoxemia following ROSC has been associated with neuronal injury, possibly due to increased production of ROS, lipid oxidation and decreased CBF [3, 49, 50]. In a randomized pilot trial, moderately elevated PaO_2_ levels (20–25 kPa) did not influence NSE levels or neurological outcome after 6 months and exposure to PaO_2_ levels ≥40 kPa following ROSC has not been investigated in a prospective randomized manner in humans [44]. However, randomized animal trials and observational human studies suggest harmful effects [12, 21, 34, 36, 37]. Our results do not support these findings entirely and randomized studies investigating increased levels of PaO_2_ in the post OHCA phase would be a possible way to further test the effect of PaO_2_ on outcome.

Our study has several limitations. Firstly, due to its observational design, the results are hypothesis generating and we cannot make causality statements. Secondly, we evaluated the most deviant PaO_2_ or PaCO_2_ values in the first 24 h after ROSC and were not able to analyze the exact exposure time-point, duration or to correct for acid-base parameters at the same time-point. Thirdly, in the statistical analyzes, our *P*-values were not significant on the 0.05 threshold level, but considering the direction of our point estimates and the width of the 95% CI’s, we cannot exclude a possible type II error and that there are associations that may have been statistically significant in a larger population [51]. We did not correct for FiO_2_ or PaO_2_/FiO_2_ ratios since FiO_2_ was not registered in the INTCAR 2.0 protocol and the PaO_2_/FiO_2_ ratio is rather an indicator for altered lung function, already accounted for by correcting for pre-existing COPD. The strengths of this study are the multicenter prospective design with 22 participating centers over two continents, a large cohort with over 2000 OHCA patients with extensive data regarding cardiac arrest characteristics and medical background and few excluded patients, as well as no missing outcome data in our primary and secondary analyses.

In summary, this study did not show an independent association of exposure to extreme PaO_2_ and PaCO_2_ values during the first 24 h after ROSC and neurological outcome at hospital discharge. The results of studies investigating exposure to extreme PaO_2_ and PaCO_2_ values vary widely and there is currently no consensus if extreme PaO_2_ or PaCO_2_ values are harmful, beneficial or innocuous to the post OHCA patient. The results of future prospective randomized studies are warranted before the existing recommendations on PaO_2_ and PaCO_2_ levels in the post OHCA phase can be revised [47, 52].

## Conclusion

In a large cohort of patients resuscitated from OHCA, exposure to extreme PaO_2_ and PaCO_2_ values in the first 24 h after ROSC occurred commonly, but was not independently associated with neurological outcome at discharge.

## Supplementary information

**Additional file 1: Table S1.** Baseline characteristics of all patients and the PaO_2_ analysis groups. **Table S2.** Baseline characteristics of all patients and the PaCO_2_ analysis groups. **Table S3.** Sensitivity analysis. Association of exposure to extreme PaO_2_ and PaCO_2_ values with poor neurological outcome (Patients with extreme PaO_2_ or PaCO_2_ value double exposure removed). **Table S4.** Association of exposure to extreme PaO_2_ and PaCO_2_ values with poor neurological long term outcome. *n* = 1850. **Table S5.** Association of exposure to extreme PaO_2_ and PaCO_2_ values with poor neurological long term outcome. Imputed values. (*n* = 2135). **Table S6.** Baseline characteristics of patients with complete PaO_2_ and PaCO_2_ values and patients with PaO_2_ or PaCO_2_ missing.

## Data Availability

The datasets used and/or analysed during the current study are available from the corresponding author on reasonable request.

## Abbreviations

PaO_2_: Arterial partial pressure of oxygen
PaCO_2_: Arterial partial pressure of carbon dioxide
TTM: Targeted temperature management
OHCA: Out-of-hospital cardiac arrest
ROSC: Return of spontaneous circulation
ROS: Reactive oxygen species
kPa: Kilopascal
CPC: Cerebral performance category
GCS: Glasgow coma scale
GCS-M: Glasgow coma scale – motor
SD: Standard deviation
IQR: Interquartile range
OR: Odds ratio
CBF: Cerebral blood flow
CDO_2_: Cerebral oxygen delivery
