# Supplementary material for: The association of partial pressures of oxygen and carbon dioxide with neurological outcome after out-of-hospital cardiac arrest: an explorative International Cardiac Arrest Registry 2.0 study
Source: Scand J Trauma Resusc Emerg Med. 2020 Jul 14;28:67. doi: 10.1186/s13049-020-00760-7 (PMC7362652; doi:10.1186/s13049-020-00760-7)
Supplement: Supplementary file 1 — Additional file 1: Table S1. Baseline characteristics of all patients and the PaO2 analysis groups. Table S2. Baseline characteristics of all patients and the PaCO2 analysis groups. Table S3. Sensitivity analysis. Association of exposure to extreme PaO2 and PaCO2 values with poor neurological outcome (Patients with extreme PaO2 or PaCO2 value double exposure removed). Table S4. Association of exposure to extreme PaO2 and PaCO2 values with poor neurological long term outcome. n = 1850. Table S5. Association of exposure to extreme PaO2 and PaCO2 values with poor neurological long term outcome. Imputed values. (n = 2135). Table S6. Baseline characteristics of patients with complete PaO2 and PaCO2 values and patients with PaO2 or PaCO2 missing. [file 13049_2020_760_MOESM1_ESM.docx]

**The association of partial pressure of oxygen and carbon dioxide with neurological outcome after out-of-hospital cardiac arrest: an explorative International Cardiac Arrest Registry 2.0 study.**

ADDITIONAL FILE

Florian Ebner MD PhD, Richard R Riker MD, Zana Haxhija MD, David B Seder MD, Teresa L May DO MS, Susann Ullén PhD, Pascal Stammet MD PhD, Karen Hirsch MD PhD, Sune Forsberg MD PhD, Allison Dupont MD, Hans Friberg MD PhD, John A McPherson MD, Eldar Søreide MD PhD, Josef Dankiewicz MD PhD, Tobias Cronberg MD PhD, Niklas Nielsen MD PhD.

| **Supplementary Table 1. Baseline characteristics of all patients and the PaO_2_ analysis groups** | | | | |
| --- | --- | --- | --- | --- |
| **Demographic characteristic** | **All patients n = 2135** | **No-exposure n = 1133** | **Hyperoxemia n = 436** | **Hypoxemia n = 422** |
| Age in years, mean (SD) | 61.09 (15.9) | 61.52 (15.54) | 59.55 (16.41) | 61.18 (16.07) |
| Male sex, n (%) | 1432 (67.1) | 787 (69.5) | 269 (61.7) | 278 (65.9) |
| **Medical history** |  |  |  |  |
| Previous myocardial infarction n (%) | 370 (17.3) | 209 (18.4) | 71 (16.3) | 74 (17.5) |
| Chronic heart failure n (%) | 367 (17.2) | 186 (16.4) | 72 (16.5) | 77 (18.2) |
| COPD n (%) | 344 (16.1) | 168 (14.8) | 85 (19.5) | 78 (18.5) |
| Cerebro vascular disease n (%) | 196 (9.2) | 115 (10.2) | 32 (7.3) | 32 (7.6) |
| Diabetes mellitus n (%) | 521 (24.4) | 263 (23.2) | 104 (23.9) | 115 (27.3) |
| Obesity n (%) | 268 (15.3) | 133 (14.5) | 53 (14.2) | 67 (18.8) |
| **Cardiac arrest characteristic** |  |  |  |  |
| Witnessed cardiac arrest n (%) | 1591 (75.6) | 859 (76.9) | 315 (72.9) | 313 (74.9) |
| Bystander CPR n (%) | 1385 (65.5) | 749 (66.8) | 292 (67.1) | 261 (62.4) |
| Bystander defibrillation n (%) | 123 (5.8) | 72 (6.4) | 27 (6.2) | 17 (4.0) |
| Initial rhythm shockable n (%) | 1022 (50.0) | 597 (54.4) | 190 (46.1) | 179 (44.3) |
| Time to ROSC (min), median (IQR) | 29 (21 - 48) | 29 (21 – 48) | 29 (19 – 48) | 34 (24 – 48) |
| **Characteristic on arrival** |  |  |  |  |
| Sedated on arrival n (%) | 437 (21.7) | 236 (22.00) | 85 (20.1) | 80 (20.00) |
| GCS Motor 1 n (%) | 1544 (79.4) | 803 (77.7) | 325 (78.5) | 320 (82.9) |
| Circulatory shock on admission n (%) | 902 (44.2) | 447 (40.9) | 203 (47.5) | 203 (49.5) |
| Admission pH, median (IQR) | 7.20 (7.10 - 7.30) | 7.21 (7.10 – 7.30) | 7.21 (7.08 – 7.30) | 7.15 (7.02 – 7.25) |
| Admission lactate, mmol/l, median (IQR) | 6.4 (3.20 - 10.2) | 5.9 (2.80 – 9.50) | 7.20 (3.7 – 10.95) | 7.30 (3.90 – 11.20) |
| Bicarbonate on admission, mmol/l, median (IQR) | 18.0 (14.5 - 21.0) | 18.8 (15.0 – 21.0) | 17.55 (14.1 – 21.0) | 17.00 (13.93 – 21.0) |

n = number, SD = standard deviation, IQR = interquartile range, % = percent, mmol/l = millimole per liter, CPR = cardio pulmonary resuscitation, ROSC = return of spontaneous circulation, COPD = chronic obstructive pulmonary disease, GCS = Glasgow coma scale, PaO_2_ = arterial partial pressure of oxygen, all % are presented as valid percent.

| **Supplementary Table 2. Baseline characteristics of all patients and the PaCO_2_ analysis groups** | | | | |
| --- | --- | --- | --- | --- |
| **Demographic characteristic** | **All patients n = 2135** | **No-exposure n = 591** | **Hypercapnemia n = 896** | **Hypocapnemia n = 683** |
| Age in years, mean (SD) | 61.09 (15.9) | 60.85 (15.90) | 61.58 (15.18) | 59.55 (16.59) |
| Male sex, n (%) | 1432 (67.1) | 435 (73.6) | 589 (65.7) | 421 (61.6) |
| **Medical history** |  |  |  |  |
| Previous myocardial infarction n (%) | 370 (17.3) | 110 (18.6) | 164 (18.3) | 110 (16.1) |
| Chronic heart failure n (%) | 367 (17.2) | 105 (17.8) | 148 (16.5) | 115 (16.8) |
| COPD n (%) | 344 (16.1) | 60 (10.2) | 208 (23.2) | 67 (9.8) |
| Cerebro vascular disease n (%) | 196 (9.2) | 58 (9.8) | 76 (8.5) | 57 (8.3) |
| Diabetes mellitus n (%) | 521 (24.4) | 126 (21.3) | 223 (24.9) | 181 (26.5) |
| Obesity n (%) | 268 (15.3) | 63 (12.7) | 136 (18.9) | 84 (14.5) |
| **Cardiac arrest characteristic** |  |  |  |  |
| Witnessed cardiac arrest n (%) | 1591 (75.6) | 450 (77.2) | 659 (74.7) | 504 (74.6) |
| Bystander CPR n (%) | 1385 (65.5) | 390 (66.2) | 576 (64.9) | 449 (66.6) |
| Bystander defibrillation n (%) | 123 (5.8) | 42 (7.1) | 49 (5.5) | 34 (5.0) |
| Initial rhythm shockable n (%) | 1022 (50.0) | 321 (57.0) | 396 (45.3) | 340 (51.9) |
| Time to ROSC (min), median (IQR) | 29 (21.0 – 48.0) | 29 (19.0 – 47.50) | 29 (21.0 – 46.0) | 32 (21.0 – 48.0) |
| **Characteristic on arrival** |  |  |  |  |
| Sedated on arrival n (%) | 437 (21.7) | 131 (23.4) | 180 (20.9) | 134 (20.7) |
| GCS Motor 1 n (%) | 1544 (79.4) | 389 (72.2) | 689 (83.4) | 523 (82.4) |
| Circulatory shock on admission n (%) | 902 (44.2) | 231 (40.6) | 404 (46.4) | 315 (47.5) |
| Admission pH, median (IQR) | 7.20 (7.10 - 7.30) | 7.24 (7.15 – 7.32) | 7.15 (7.02 – 7.24) | 7.22 (7.10 – 7.31) |
| Admission lactate, mmol/l, median (IQR) | 6.4 (3.20 - 10.2) | 5.38 (2.8 – 9.5) | 7.07 (3.6 – 10.8) | 6.20 (3.1 – 10.4) |
| Bicarbonate on admission, mmol/l, median (IQR) | 18.0 (14.5 - 21.0) | 18.6 (15.0 – 21.0) | 18.8 (14.1 – 22.0) | 17.80 (14.0 – 20.1) |

n = number, SD = standard deviation, IQR = interquartile range, % = percent, mmol/l = millimole per liter, CPR = cardio pulmonary resuscitation, ROSC = return of spontaneous circulation, COPD = chronic obstructive pulmonary disease, GCS = Glasgow coma scale, PaCO_2_ = arterial partial pressure of carbon dioxide, all % are presented as valid percent.

| **Supplementary Table 3. Sensitivity analysis. Association of exposure to extreme PaO_2_ and PaCO_2_ values with poor neurological outcome (Patients with extreme PaO_2_ or PaCO_2_ value double exposure removed)** | | | |
| --- | --- | --- | --- |
|  | **OR** | **95% CI** | **p-value** |
| Hyperoxemia versus PaO_2_ no-exposure | 1.44 | 0.97 - 2.14 | 0.07 |
| Hyperoxemia versus no-hyperoxemia | 1.37 | 0.94 - 2.02 | 0.11 |
| Hypoxemia versus PaO_2_ no-exposure | 1.36 | 0.91 - 2.03 | 0.13 |
| Hypoxemia versus no-hypoxemia | 1.26 | 0.86 - 1.86 | 0.24 |
| Hypercapnemia versus PaCO_2_ no-exposure | 0.83 | 0.58 - 1.18 | 0.29 |
| Hypercapnemia versus no-hypercapnemia | 0.82 | 0.59 - 1.12 | 0.21 |
| Hypocapnemia versus PaCO_2_ no-exposure | 1.30 | 0.88 - 1.93 | 0.19 |
| Hypocapnemia versus no-hypocapnemia | 1.25 | 0.89 - 1.77 | 0.20 |

OR = odds ratio, 95% CI = 95% confidence interval, PaO_2_= arterial partial pressure of oxygen, PaCO_2_= arterial partial pressure of carbon dioxide. Hyperoxemia = PaO_2_ >40 kPa, Hypoxemia = PaO_2_ <8.0 kPa, Hypercapnemia = PaCO_2_ >6.7 kPa, Hypocapnemia = PaCO_2_ <4.0 kPa. PaO_2_ no-exposure = 8.0-40 kPa, PaCO_2_ no-exposure = 4.0-6.7 kPa. Double exposure = hyperoxemia and hypoxemia or hypercapnemia and hypocapnemia.

| **Supplementary Table 4. Association of exposure to extreme PaO_2_ and PaCO_2_ values with poor neurological long term outcome. n = 1850** | | | |
| --- | --- | --- | --- |
| **Analysis** | **OR** | **95% CI** | **P-Value** |
| Hyperoxemia versus PaO_2_ no-exposure | 1.29 | 0.85 – 1.95 | 0.23 |
| Hyperoxemia versus no-hyperoxemia | 1.25 | 0.84 – 1.87 | 0.28 |
| Hypoxemia versus PaO_2_ no-exposure | 1.21 | 0.79 – 1.84 | 0.38 |
| Hypoxemia versus no-hypoxemia | 1.15 | 0.77 – 1.73 | 0.49 |
| Hypercapnemia versus PaCO_2_ no-exposure | 0.97 | 0.67 – 1.42 | 0.89 |
| Hypercapnemia versus no-hypercapnemia | 0.89 | 0.64 – 1.24 | 0.49 |
| Hypocapnemia versus PaCO_2_ no-exposure | 1.35 | 0.90 – 2.01 | 0.14 |
| Hypocapnemia versus no-hypocapnemia | 1.29 | 0.92 – 1.82 | 0.14 |

OR = odds ratio, 95% CI = 95% confidence interval, PaO_2_= arterial partial pressure of oxygen, PaCO_2_= arterial partial pressure of carbon dioxide. Hyperoxemia = PaO_2_ >40 kPa, Hypoxemia = PaO_2_ <8.0 kPa, Hypercapnemia = PaCO_2_ >6.7 kPa, Hypocapnemia = PaCO_2_ <4.0 kPa. PaO_2_ no-exposure = 8.0-40 kPa, PaCO_2_ no-exposure = 4.0-6.7 kPa.

| **Supplementary Table 5. Association of exposure to extreme PaO_2_ and PaCO_2_ values with poor neurological long term outcome. Imputed values. (n = 2135)** | | | |
| --- | --- | --- | --- |
| **Analysis** | **OR** | **95% CI** | **P-Value** |
| Hyperoxemia versus PaO_2_ no-exposure | 1.28 | 0.89 – 1.85 | 0.18 |
| Hyperoxemia versus no-hyperoxemia | 1.18 | 0.83 – 1.68 | 0.35 |
| Hypoxemia versus PaO_2_ no-exposure | 1.23 | 0.85 – 1.78 | 0.28 |
| Hypoxemia versus no-hypoxemia | 1.15 | 0.81 – 1.65 | 0.43 |
| Hypercapnemia versus PaCO_2_ no-exposure | 0.91 | 0.65 – 1.28 | 0.59 |
| Hypercapnemia versus no-hypercapnemia | 0.87 | 0.64 – 1.16 | 0.34 |
| Hypocapnemia versus PaCO_2_ no-exposure | 1.32 | 0.92 – 1.89 | 0.13 |
| Hypocapnemia versus no-hypocapnemia | 1.25 | 0.92 – 1.69 | 0.15 |

OR = odds ratio, 95% CI = 95% confidence interval, PaO_2_= arterial partial pressure of oxygen, PaCO_2_= arterial partial pressure of carbon dioxide. Hyperoxemia = PaO_2_ >40 kPa, Hypoxemia = PaO_2_ <8.0 kPa, Hypercapnemia = PaCO_2_ >6.7 kPa, Hypocapnemia = PaCO_2_ <4.0 kPa, PaO_2_ no-exposure = 8.0-40 kPa, PaCO_2_ no-exposure = 4.0-6.7 kPa.

| **Supplementary Table 6. Baseline characteristics of patients with complete PaO_2_ and PaCO_2_ values and patients with PaO_2_ or PaCO_2_ missing.** | | |
| --- | --- | --- |
| **Demographic characteristic** | **PaO_2_ and PaCO_2_ complete (n=1891)** | **PaO_2_ or PaCO_2_ missing (n=244)** |
|  | **Value** | **Value** |
| Age in years, mean (SD) | 61.01 (15.8) | 61.7 (16.5) |
| Male sex, n (%) | 1270 (67.2) | 162 (66.4) |
| **Medical history** |  |  |
| Previous myocardial infarction n (%) | 342 (18.1) | 28 (11.5) |
| Chronic heart failure n (%) | 321 (17.0) | 46 (18.9) |
| COPD n (%) | 306 (16.2) | 38 (15.6) |
| Cerebro vascular disease n (%) | 171 (9.0) | 25 (10.2) |
| Diabetes mellitus n (%) | 459 (24.3) | 62 (25.4) |
| Obesity n (%) | 241 (15.4) | 27 (14.5) |
| **Cardiac arrest characteristic** |  |  |
| Witnessed cardiac arrest n (%) | 1419 (75.9) | 172 (72.9) |
| Bystander CPR n (%) | 1240 (66.2) | 145 (60.7) |
| Bystander defibrillation n (%) | 113 (6.0) | 10 (4.1) |
| Initial rhythm shockable n (%) | 926 (50.9) | 96 (42.7) |
| Time to ROSC (min), median (IQR) | 29 (21 – 48) | 32 (19 – 53) |
| **Characteristic on arrival** |  |  |
| Sedated on arrival, n (%) | 388 (21.6) | 49 (22.9) |
| GCS Motor 1, n (%) | 1371 (78.9) | 173 (83.2) |
| Circulatory shock on admission, n (%) | 805 (44.0) | 97 (46) |
| Admission pH, median (IQR) | 7.2 (7.1 – 7.3) | 7.2 (7.0 – 7.3) |
| Admission lactate, mmol/l median (IQR) | 6.2 (3.1 – 10.0) | 8.0 (4.1 – 11.3) |
| Bicarbonate on admission, mmol/l, median (IQR) | 18 (14.5 – 21.0) | 18 (15.0 – 22.0) |

n = number, SD = standard deviation, IQR = interquartile range, % = percent, mmol/l = millimole per liter, CPR = cardio pulmonary resuscitation, ROSC = return of spontaneous circulation, COPD = chronic obstructive pulmonary disease, GCS = Glasgow coma scale, PaO_2_= arterial partial pressure of oxygen, PaCO_2_= arterial partial pressure of carbon dioxide, all % are presented as valid percent.
